# ESR Essentials: pelvic floor imaging—practice recommendations by the European Society of Urogenital Radiology

**DOI:** 10.1007/s00330-026-12706-1

**Published:** 2026-07-01

**Authors:** Mohamed A. Abdelatty, Aleksandra Stankiewicz, Francesca Maccioni, Rebeca Mirón Mombiela, Basma Amer, Sandra Sousa, Sonya Snape, Carlos Carnelli, Rita Lucas, Geertje Balk-Roos, Andelib Babatürk, Rania F. El Sayed

**Affiliations:** 1 Pelvic Floor Imaging Unit and Research Lab, Department of Radiology, Kasr Al-Ainy Hospitals, Cairo University, Cairo, Egypt; 2 Department of Radiology, University Hospitals of North Midlands NHS Trust, Stoke-on-Trent, UK; 3 Department of Radiological, Oncological and Pathological Sciences, Policlinico Umberto I Hospital, Sapienza University, Rome, Italy; 4 Department of Radiology, Herlev and Gentofte Hospital, Herlev, Denmark; 5 Institute for Clinical Medicine, University of Copenhagen, Copenhagen, Denmark; 6 Radiology Department, Hospital Lusíadas Lisboa, Lisboa, Portugal; 7 Department of Radiology, University Hospitals Dorset, Dorset, UK; 8 Department of Radiology, Asistencial Médica de Maldonado, Punta del Este, Uruguay; 9 Department of Radiology and Nuclear Medicine, Luzerner Kantonsspital, University Teaching and Research Hospital, University of Lucerne, Luzern, Switzerland; 10 Department of Radiology, Ankara Güven Hospital, Ankara, Turkey

**Keywords:** Pelvic floor disorders, Defecography, Magnetic resonance imaging, Fluoroscopy, Ultrasonography

## Abstract

**Abstract:**

Pelvic floor dysfunction is a common and underdiagnosed condition affecting urinary, gastrointestinal, and sexual function. Imaging plays a pivotal role in diagnosis and treatment planning, yet approaches to technique, interpretation, and reporting remain inconsistent. This article, authored by members of the ESUR Pelvic Floor Working Group, provides practical recommendations for the use of fluoroscopic defecography, MR defecography, and pelvic floor ultrasound, including standardized protocols, key measurements, and a structured, compartment-based interpretation strategy. We detail the imaging features of anterior, middle, and posterior compartment disorders and highlight common pitfalls. Implementing these recommendations by adopting the suggested protocols and reporting approach in routine practice will help ensure consistent, clinically relevant pelvic floor assessments.

**Key Points:**

- Pelvic floor dysfunction frequently involves multiple compartments, and MRI provides a detailed structural and functional assessment of all pelvic compartments.
- Fluoroscopic defecography remains the mainstay for the evaluation of defecatory disorders.
- Pelvic floor ultrasound is increasingly valuable for triaging pelvic floor dysfunction and for detailed assessment of pelvic floor anatomy and anal sphincter integrity.

## Key recommendations

- MR defecography should be used for comprehensive assessment of pelvic floor dysfunction, particularly in complex multicompartment disease or after prior surgery, provided high-quality images are obtained with adequate patient cooperation (level of evidence: high).
- Conventional fluoroscopic defecography is a valuable tool in evaluating defecatory dysfunction despite the widespread use of MR defecography. It can be used to provide crucial information when an MRI is inconclusive and may yield more representative functional information (level of evidence: high).
- Endoanal ultrasound is the preferred modality for assessing anal sphincter integrity, offering superior sensitivity in detecting internal anal sphincter tears and comparable sensitivity to MRI in external anal sphincter tears (level of evidence: high).

## Introduction and pelvic floor dysfunction

Pelvic floor dysfunction (PFD) encompasses a broad spectrum of symptoms, reflecting the pelvic floor’s role in supporting sexual, urinary, and gastrointestinal function. To conceptualize this complexity, several classification systems have been proposed, most commonly the three-compartment model, comprising the anterior (urinary bladder and urethra), middle (uterus and vagina), and posterior (anorectum) compartments [1]. However, the pelvic floor rarely behaves in neatly separated compartments, and patients frequently demonstrate multicompartmental involvement despite apparently localized symptoms. In some cases, dysfunction in one compartment can even recruit another over time, with chronic straining serving as a classic example. Consequently, many clinicians regard the pelvic floor as a single integrated functional unit that should be assessed holistically, irrespective of symptom origin [2].

### Common pelvic floor referrals for radiological assessment

Urinary incontinence refers to involuntary urine leakage and is classified into stress, urge, and mixed types. Stress urinary incontinence (SUI) results from increased intra-abdominal pressure due to urethral sphincter weakness or urethral hypermobility, whereas urge incontinence is associated with detrusor overactivity and a sudden urge to void. Mixed incontinence combines features of both [3].

Pelvic organ prolapse (POP) serves as an umbrella term describing the descent of one or more organs onto the anterior vaginal wall (e.g., bladder), posterior vaginal wall (rectocele or cul-de-sac hernia), or apical (vaginal cuff or hysterectomy stump). Symptoms vary and may include perineal bulging, a sensation of incomplete evacuation, pelvic heaviness, or back pain [4].

Chronic pelvic pain and dyspareunia are defined as persistent pelvic discomfort and painful sexual intercourse and may arise from multiple pelvic conditions; however, in this review, emphasis is placed on pelvic floor disorders such as rectocele, cystocele, and enterocele. These symptoms often appear alongside urinary or defecatory complaints, but they are quite frequently the very issues that lead patients to seek clinical help [4].

Obstructed defecation syndrome (ODS) represents a form of functional constipation characterized by impaired rectal evacuation despite an urge to defecate. Hallmark features include infrequent bowel movements, excessive straining, incomplete emptying, a sensation of obstruction, and, in some cases, the need for digital rectal evacuation [5, 6].

Fecal incontinence (FI) is defined as the involuntary loss of stool and may be classified as urge, passive, or nocturnal. Patients are often referred for imaging to assess anal sphincter integrity; however, continence depends on a coordinated pelvic support system, and sphincters may appear normal despite clinically evident FI. Imaging, therefore, remains valuable for identifying contributory mechanisms and guiding management [5, 6].

### Imaging modalities for investigating pelvic floor dysfunction

**Fig. 1:**
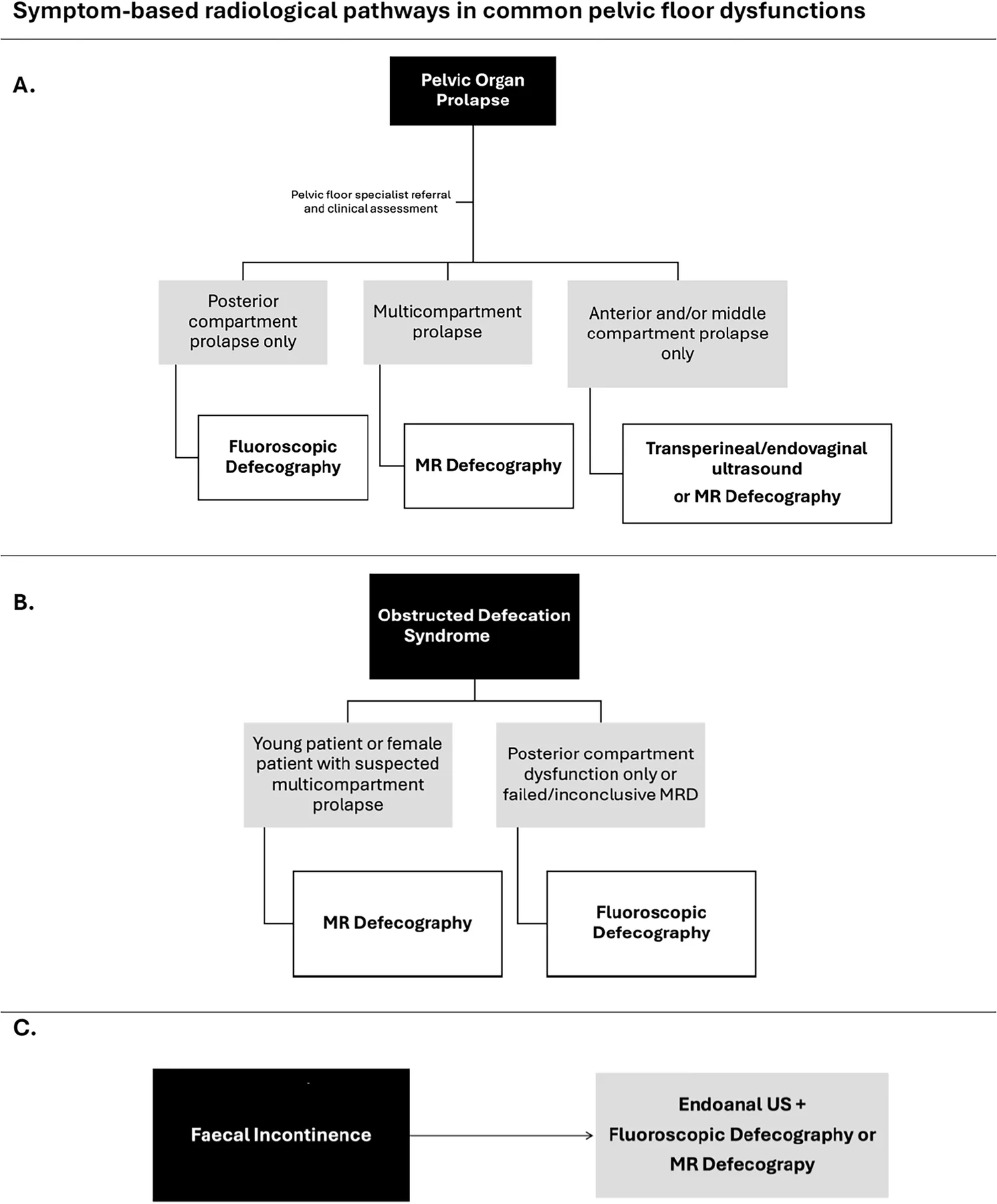
Flowchart of common symptom-based radiological pathways in PFD patients (**A**) POP (**B**) ODS and (**C**) FI

The choice of the most appropriate imaging depends on several factors, including patient compliance and local availability, and often requires the use of more than one modality (Fig. 1). Depending on availability, fluoroscopic defecography (FD) or MR defecography (MRD) is generally the first-line imaging option for patients with defecatory symptoms. In patients with chronic pelvic pain, dyspareunia, or suspected pelvic organ prolapse, MRD can provide a comprehensive one-stop shop assessment [6–8].

## Fluoroscopic defecography

Fluoroscopic defecography (FD) or proctogram is a widely used imaging technique for evaluating pelvic floor function. It is commonly employed to assess defecatory dysfunction and provides valuable insights into the functional integrity of the posterior compartment.

There is no consensus regarding patient preparation for FD. Some authors recommend a cleansing enema on the day of the examination, while others advocate retaining fecal matter, as it may better simulate physiological conditions. Moreover, practices may vary between centers, with some performing double- or triple-contrast studies that include, in addition to rectal opacification, small bowel and vaginal contrast (Supplementary Table S1) [9]. Bladder opacification is not routinely performed. When the evaluation of all three compartments is desired, MRI is currently the preferred alternative.

Adequate patient instruction and coaching are essential for accurately assessing pelvic floor muscle (PFM) dynamics, changes in the anorectal angle (ARA), and perineal descent. The patient must be cooperative and able to follow instructions, including performing the Valsalva maneuver and PFM contraction. Maintaining patient dignity, privacy, and demonstrating appropriate bedside manners are essential to ensure patient comfort and cooperation [9, 10].

The examination is performed in the sitting position, making it an ideal functional study to simulate defecation. Nonetheless, it does not represent true physiologic defecation, and the use of this term should be avoided. FD findings are derived from the voluntary expulsion of a contrast paste rather than the natural passage of stools. In contrast, normal defecation is a complex process influenced by gut motility, stool consistency, neural regulation of the anal canal and pelvic floor, rectal compliance and sensitivity, and lastly the coordinated function of the anal sphincters and PFM [9].

A standard FD should include a minimum set of images to allow comprehensive functional assessment of the posterior compartment. These typically consist of pre-evacuation, evacuation-phase, and post-evacuation views. The evacuation sequence is essential for evaluating dynamic changes in ARA and rectal emptying, as well as for detecting structural abnormalities such as rectocele, intussusception, or prolapse (Table 1). Finally, a post-evacuation image documents the completeness of rectal emptying and the final position of pelvic structures, confirming whether contrast retention or persistent prolapse is present [11, 12].

**Table 1:** Minimum reporting dataset for fluoroscopic defecography and MR defecography

Finding	Definition and how to assess?	Fluoroscopic defecography (FD)	MR defecography (MRD)
Initiation of evacuation	Patient was able to initiate evacuation when instructed. If delayed, mention the time required to begin emptying.		
Rate and completeness of emptying	How much of the contrast was evacuated in the first minute? Completeness of rectal emptying should be graded as complete, partial, or absent after 1 min and by the end of the evacuation.		
Changes in ARA	Report relaxation or contraction of the puborectalis muscle. At rest, ARA is near a right angle, becoming narrower (acute) with PFM contraction and wider (obtuse) during straining and evacuation.		
ARJ descent	Measured compared to its initial position before evacuation attempts. Pathological if > 4 cm below PCL.	If PCL cannot be identified confidently on FD, other bony landmarks can be used (e.g., ischial spine) or even the commode.	PCL can be drawn on the midsagittal images, extending from the inferior aspect of the pubic symphysis to the last transverse coccygeal joint.
Rectocele	Anterior bulging of the rectal wall beyond its expected boundary. - Abnormal if > 2 cm - Graded according to the rule of 2: • Grade I: 2 cm • Grade II: 2–4 cm • Grade III: > 4 cm - Mention if trapping or non-trapping by the end of evacuation		
Rectal intussusception	Invagination of the rectal wall upon itself within the rectal or the anal lumen or external prolapse. - The Oxford grading system is recommended: • Grade I (high recto-rectal): descends just proximal to the upper limit of the rectocele. • Grade II (low recto-rectal): descends into the rectocele but not the anal canal. • Grade III (high recto-anal): descends into the upper sphincter or anal canal. • Grade IV (low recto-anal): descends into the lower sphincter or anal canal. • Grade V (extra-anal rectal prolapse): protrudes beyond the anal canal.		
		
Cul-de-sac hernias	Herniation of peritoneal fat, small bowel, or sigmoid colon below the PCL within the rectovaginal/rectovesical space. - Graded according to the rule of 3: • Grade I: 3 cm • Grade II: 3–6 cm • Grade III: > 6 cm		Enterocele
Cystocele	Abnormal descent of the urinary bladder below the PCL -Minor descent of 1–2 cm below the PCL is normal - Graded according to the rule of 3: • Grade I: 3 cm • Grade II: 3–6 cm • Grade III: > 6 cm	Not directly assessed but can be inferred by the “dish” appearance of the opacified vagina.	
Uterine descent	Abnormal descent of the cervix/vaginal stump below the PCL - Graded according to the rule of 3: • Grade I: 3 cm • Grade II: 3–6 cm • Grade III: > 6 cm	Not directly assessed, but the use of vaginal contrast can infer abnormal descent.	

## MRI of the pelvic floor or MR defecography

MRI has become a key imaging technique for assessing the pelvic floor due to its excellent soft-tissue contrast and the ability to perform real-time dynamic evaluation of the three pelvic compartments without radiation exposure. This technique enables detailed visualization of pelvic floor anatomy and function during PFM contraction, straining, and evacuation. Although the physiological position during defecation can only be reproduced in the upright scanners, which are not widely available, it has been reported that scanning in the supine position can provide similar information [13, 14].

### Technical considerations

MRD is typically performed using a 1.5-T scanner with a phased-array coil. The rectum should be opacified with ultrasound gel, and the patient should be examined in a supine position with their knees elevated on a pillow [15, 16]. Vaginal gel may improve the assessment of vaginal vault prolapse following hysterectomy. A preparatory enema or other bowel cleansing is not mandatory but recommended to reduce the patient’s discomfort and because large volume residual fecal matter can hinder gel evacuation, although some practitioners believe it is useful to mimic defecation [17]. Preparing the patient in advance on how to execute squeezing, straining, and evacuation maneuvers is crucial. During the evacuation phase, the patient should be instructed to repeat the evacuation process until the rectum is emptied. Using diapers to protect the patient is recommended to prevent soiling.

#### Imaging technique

MR defecography should include both static and dynamic imaging phases. The static phase is performed to assess the entire pelvis and is acquired in the sagittal, axial, and coronal planes using fast T2-weighted sequences. High-resolution T2-weighted images may be required for detailed evaluation of fascial or muscular defects involving the pelvic floor muscles, pelvic ligaments, and anal sphincter. The dynamic phase represents the functional and most clinically relevant component of MR defecography. Dynamic midsagittal imaging is performed using single-shot T2-weighted or balanced steady-state gradient sequences (e.g., TRUE-FISP, FIESTA, or BTFE) during defecation and should generally be repeated three times, regardless of rectal emptying. If rectal evacuation is incomplete, additional repetitions are recommended until emptying occurs. Additional dynamic midsagittal acquisitions during squeezing and Valsalva maneuver may also be obtained prior to the defecation phase, as they can provide complementary functional information [16, 17].

**Fig. 2:**
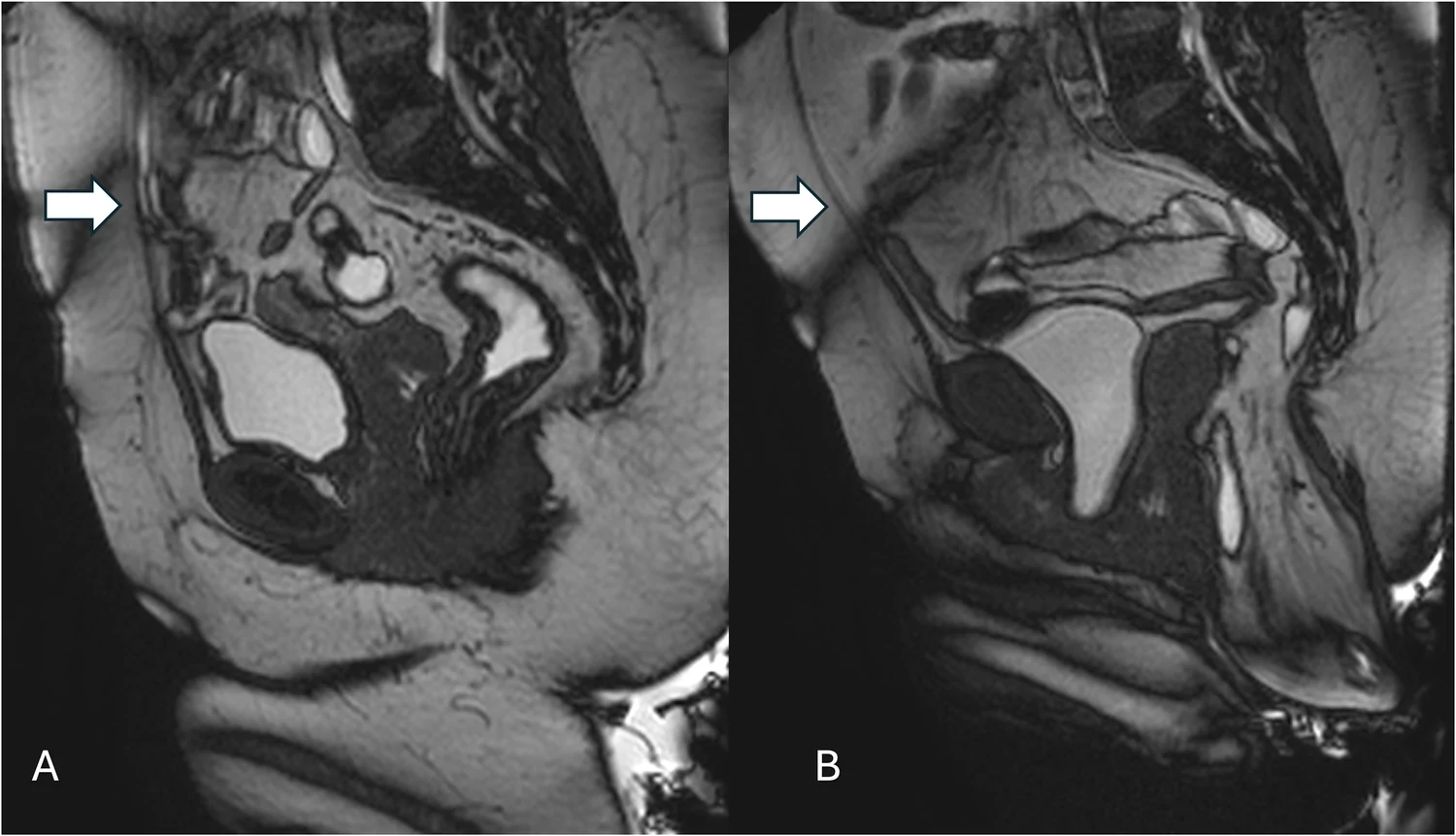
MRD images in a woman with pelvic organ prolapse (**A**) at rest and (**B**) during evacuation. Note the anterior bowing of the anterior abdominal wall muscles (arrows), confirming adequate patient effort. Evacuation was not repeated, as the initial attempt was diagnostic, given the evident global pelvic organ prolapse and associated abdominal wall muscle contraction

**Fig. 3:**
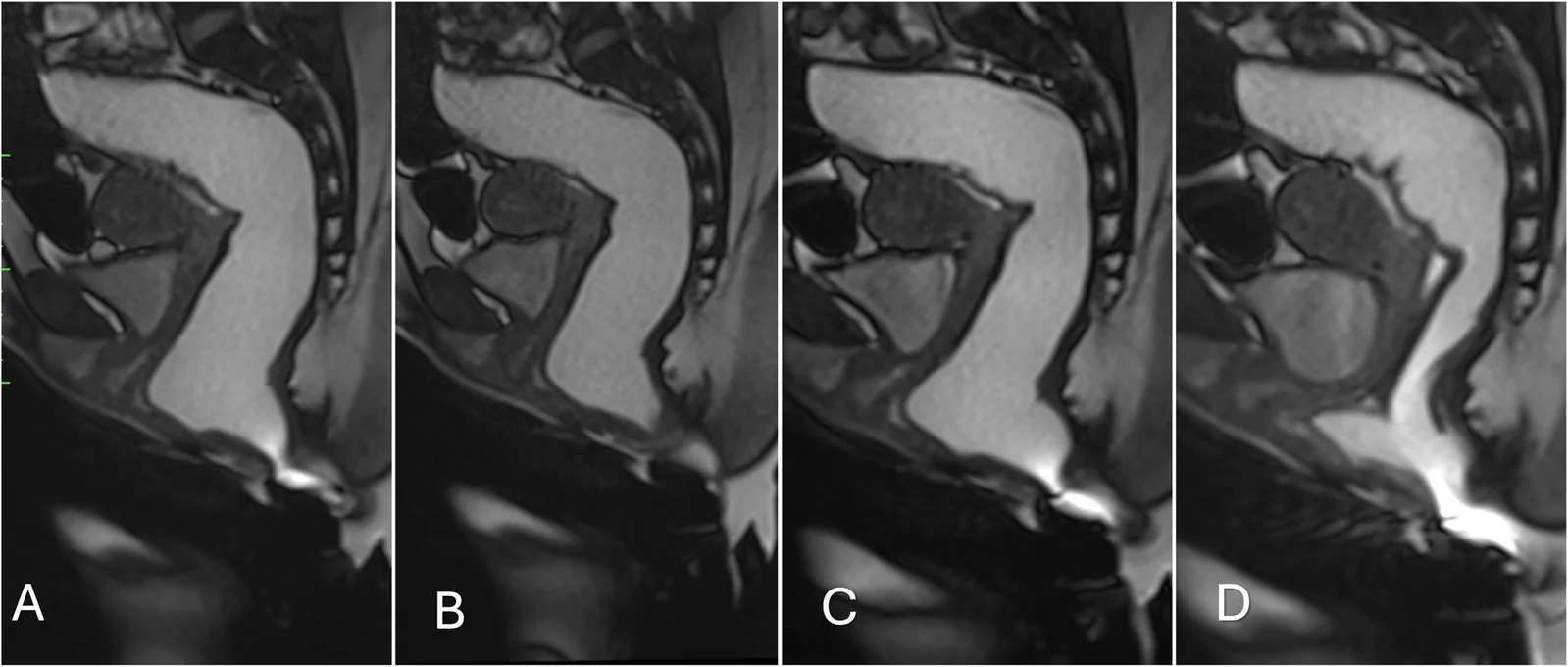
Sagittal FSP T2WI during repeated evacuation trials, demonstrating how the diagnosis evolves with each attempt and its impact on management. **A**–**C** Consecutive MRD images with the first three attempts show an anterior rectocele with a tight distal anal canal, whereas only the final evacuation (**D**) reveals the underlying recto-rectal intussusception. This case outlines the importance of repeating the evacuation phase at least three times in MRD to show hidden functional disorders

The study can be deemed diagnostic if the distinct “bowing” movement of the abdominal wall is observed during straining (Fig. 2), ideally with gel seen traversing the anal canal lumen [16]. The evacuation sequence should be repeated until the rectum is fully emptied to rule out rectal intussusception (Fig. 3). If there is no evacuation of the rectal contents after 3 attempts, or if the evacuation time is delayed, defecatory dyssynergia or anismus should be considered; however, MRI embarrassment could also be the cause for the patient’s inability to propel the gel.

### Interpretation of MRD

Pelvic organ descent is measured relative to the pubococcygeal line (PCL), drawn on the midsagittal plane from the inferior aspect of the pubic symphysis to the last transverse coccygeal joint. Reference points include the bladder base (anterior), cervix or vaginal vault (middle), and anorectal junction (posterior). Reporting organ movement relative to resting position provides greater clinical value than grading alone [16, 17].

**Fig. 4:**
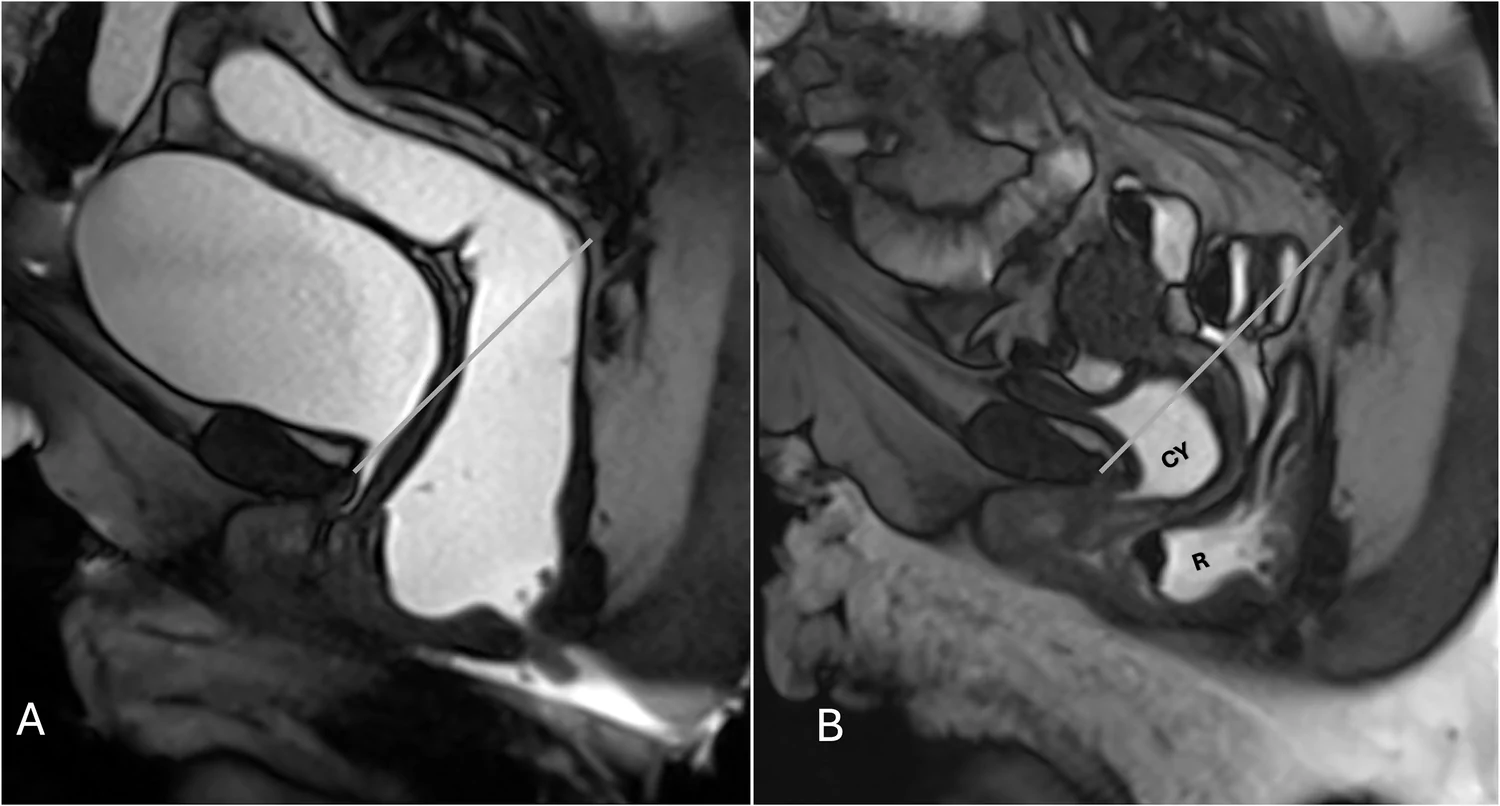
MRD images from a female patient with ODS illustrating the importance of selecting the correct phase for organ-descent measurements, as well as the importance of obtaining several evacuation attempts. **A** No bladder base or uterine descent below the PCL is seen at the first evacuation attempt. Instead, after rectal evacuation and partial bladder emptying (**B**), marked organ descent below the PCL (line) becomes evident with a cystocele (CY) as well as a rectal intussusception and a larger anterior rectocele (R), making this the appropriate phase for accurate compartment grading

The commonly applied “rule of three” defines significant anterior or middle compartment descent as greater than 3 cm below the PCL, while posterior compartment descent is considered significant beyond 4 cm during evacuation, reflecting greater physiological mobility [18, 19]. Additional supportive parameters include the ARA, levator hiatus width, and levator plate configuration can also be used to assess PFM dynamics [20]. Posterior compartment descent should be assessed during evacuation, while anterior and middle compartment descent are ideally measured after rectal emptying (Fig. 4) [18].

### Reporting tips and common pitfalls in MRD

Reports should include anatomic comments on the integrity of pelvic floor muscles and anal sphincters at rest. Clinicians rely on radiologists to identify the underlying mechanism of pelvic floor dysfunction, such as muscle weakness versus fascial defects, to guide management. Functional pelvic floor sagging may be managed conservatively with physiotherapy, whereas discrete structural defects typically require surgical repair [21]. Radiologists should comment on the adequacy of straining and avoid overinterpreting physiological descent, minor rectoceles, bladder base descent, or small mucosal intussusceptions, which may represent normal findings (Table 1) [22].

### MR defecography versus fluoroscopic defecography (Table 2)

**Fig. 5:**
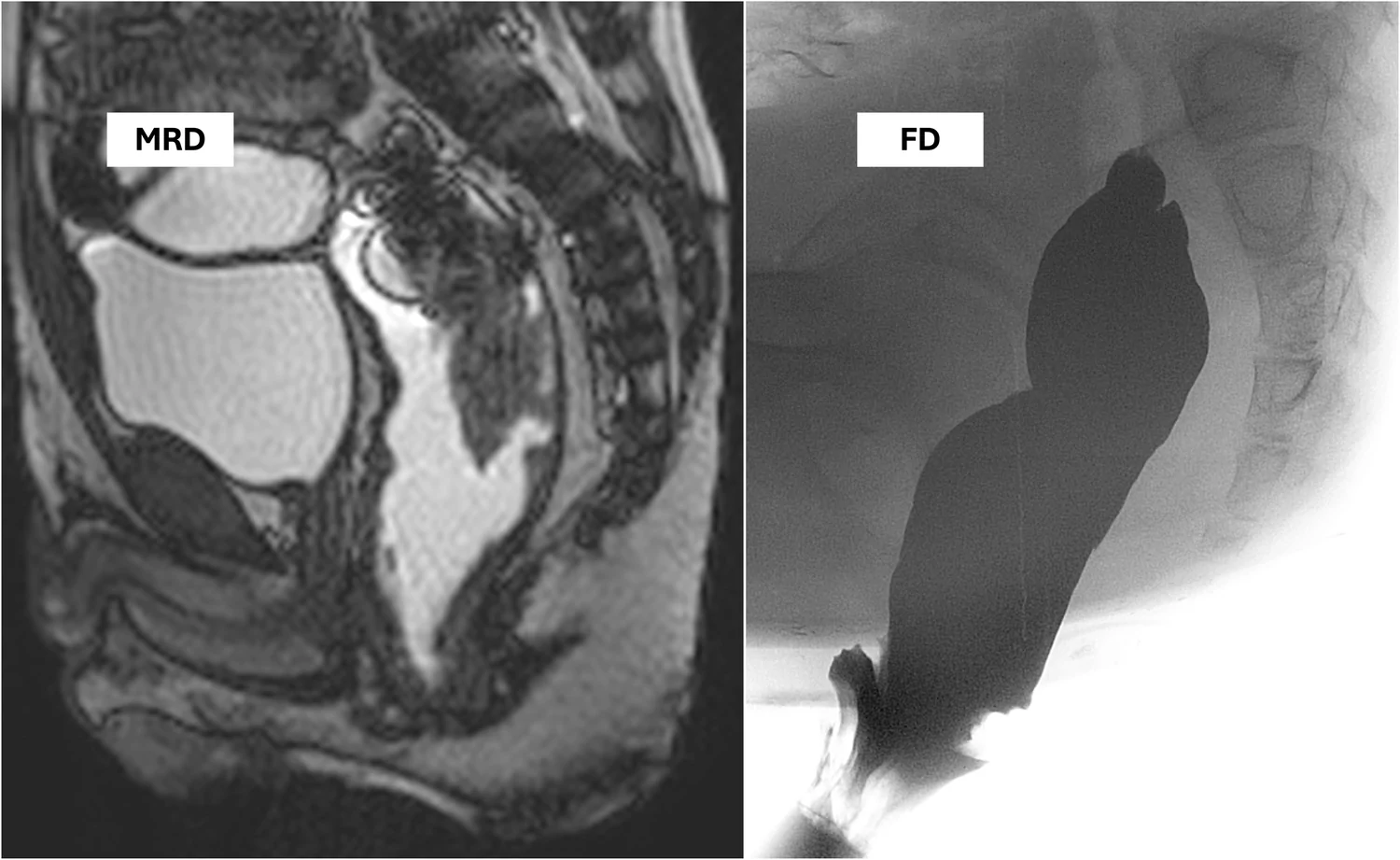
An 18-year-old male with ODS and suspected rectal prolapse. MRD was initially performed to avoid radiation exposure from FD; however, evacuation was not achieved during MRD. This was attributed to MRI-related embarrassment rather than true dyssynergia. Subsequent FD demonstrated high-grade rectal intussusception. Note that rectal cleansing was not achieved during MRD which may result in potential embarrassment. Hence, rectal cleansing prior to MRD may facilitate emptying of rectal contents

**Fig. 6:**
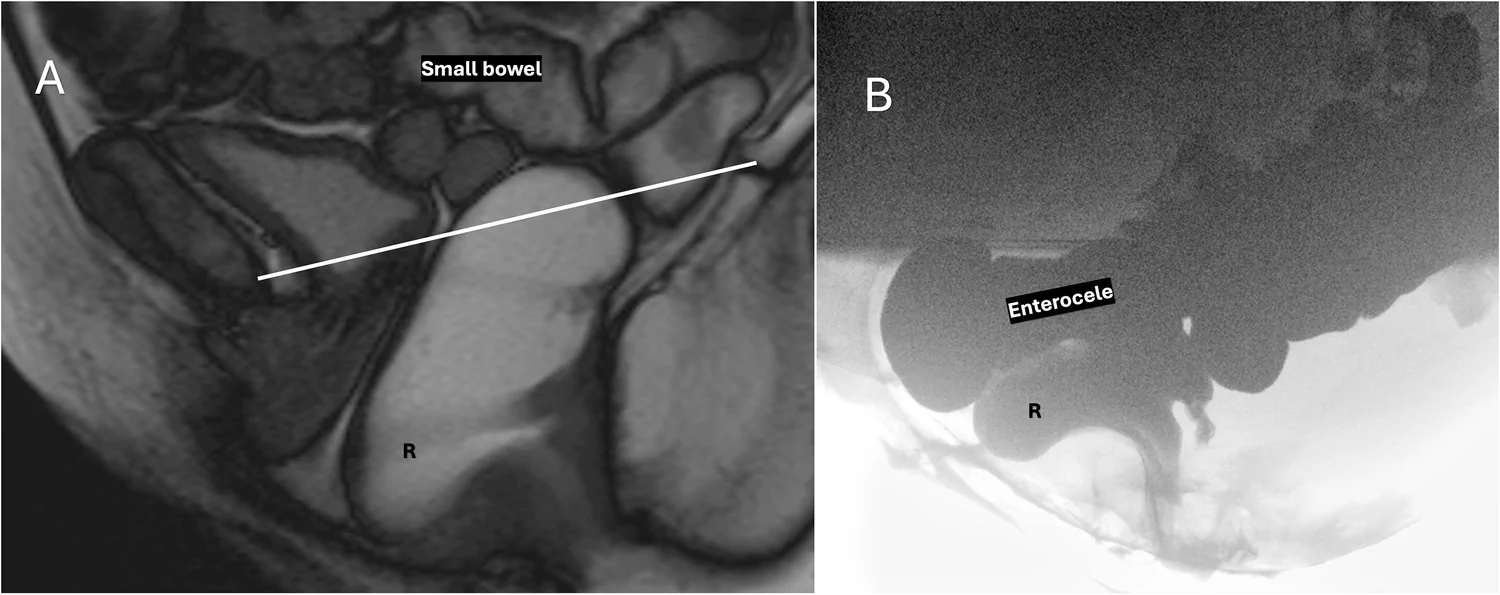
**A** MRD demonstrates an anterior rectocele (R) without evidence of enterocele in a 72-year-old patient presenting with a vaginal bulge during defecation. However, her symptoms did not improve after physiotherapy, and she was therefore referred for fluoroscopic assessment. **B** FD reveals a large enterocele with small bowel herniation below the PCL, which was not depicted on MRD, highlighting the importance of clinical correlation and the complementary role of FD in selected cases

MRD enables a comprehensive, radiation-free assessment of all pelvic compartments with accurate measurements and staging, and allows repeated straining for functional evaluation; on the contrary, FD is a one-shot examination, thus straining maneuvers are not usually repeated, to avoid over-exposure to radiation. FD remains useful for isolated defecatory symptoms, and in patients with MRI contraindications or claustrophobia. Additionally, when MRD is inconclusive, if the patient is unable to defecate within the confined supine setting of the MRI scanner, FD may provide more representative functional information (Fig. 5). FD also allows the direct identification of small bowel loops descending into the rectovaginal space during straining when oral contrast is administered ahead of the examination (Fig. 6). Accordingly, FD may offer a more precise evaluation of the severity of posterior compartment abnormalities, particularly when MRD does not fully account for the patient’s symptoms [23]. However, patients report higher overall experience scores for MRI, possibly due to greater privacy, although evacuation is often more difficult in the scanner [23].

**Table 2:** Comparison between fluoroscopic defecography and MR defecography

Feature	Fluoroscopic defecography (FD)	MR defecography (MRD)
Imaging modality	Real-time fluoroscopy	MRI with static and dynamic sequences
Soft-tissue contrast	Limited	Excellent (muscles, fascia, ligaments, sphincters)
Patient position	Seated (more physiologic defecation posture)	Supine
Radiation exposure	Yes	No
Patient tolerance	Better in claustrophobic patients, lower patient satisfaction compared to MRD	Limited by claustrophobia or anxiety. Overall, better patient satisfaction and more privacy.
Typical clinical role	Isolated defecatory dysfunction, or inconclusive/equivocal MRD	Comprehensive pelvic floor evaluation
Contraindications	No absolute contraindications, apart from avoiding irradiation in younger patients	Implanted devices, claustrophobia, and severe anxiety
Dynamic assessment of evacuation	Excellent real-time visualization	Excellent, real-time, but more dependent on patient cooperation
Assessment of evacuation completeness	Highly reliable but ‘one-shot’ examination (not repeatable)	Reliable, less physiological, and can be repeated several times
Posterior compartment evaluation	Very accurate, but poor anatomic details	Accurate, better anatomic details and reference lines
Anterior and middle compartment evaluation	Limited (unless additional contrast materials are used)	Superior multicompartment assessment
Detection of cul-de-sac hernias	Limited differentiation of bowel contents, but more conclusive than MRD due to positioning.	Superior in distinguishing between enterocele, sigmoidocele, and peritoneocele
Quantification of organ descent and assessment of pelvic floor motion	Reliable, standardized reference lines	Standardized reference lines (e.g., PCL, HMO system), with clearer anatomical landmarks
Impact on surgical planning	High, changes management in ~ 50% of cases	High, especially for multicompartment disease

## Ultrasound of the pelvic floor

Ultrasound is widely available, cost-effective, and free of ionizing radiation, offering dynamic evaluation of pelvic floor anatomy and function [3]. Pelvic floor ultrasound includes TPUS, EVUS, and EAUS, each with distinct characteristics summarized in Table 3. No vaginal or rectal contrast is required, and the urinary bladder should be comfortably full [3].

**Table 3:** Ultrasound of the pelvic floor approaches and techniques

	Transperitoneal ultrasound (TPUS)		Endovaginal ultrasound (EVUS)		Endoanal ultrasound (EAUS)
	2D/dynamic	3D/4D	2D/dynamic	3D	2D/3D
Position	Dorsal lithotomy		Dorsal lithotomy		Prone or left lateral decubitus
Plane	Midsagittal	Axial	Midsagittal		Endocavitary 360° probe (6–16 MHz)
Probe type and frequency	Convex probe (1.6–6 MHz)	Volumetric probe 2–8 MHz	Biplane mechanical probe 4–14 MHz	3D biplane Mechanical 360° probe (4–14 MHz)	
Structures assessed	Bladder/urethra, vagina, anorectum	Levator hiatus, levator ani muscles	Urethra, anorectum	Levator hiatus, levator ani muscles	Anal sphincters
	P, pubic bone; U, urethra; V, vagina; A, anal canal; PB, perineal body; PR, puborectalis; LAM, levator ani muscles; R, rectum.				

### Transperineal ultrasound

Transperineal ultrasound (TPUS) is performed with the ultrasound transducer positioned on the perineum, labia, or vaginal introitus to visualize all anatomical structures between the posterior margin of the symphysis pubis and the anterior margin of the coccyx. Images are acquired in the midsagittal plane at rest, during maximal Valsalva maneuver, and during PFM contraction (squeeze) to dynamically define the position and anatomical relationships of pelvic structures. Dynamic TPUS enables identification of cystocele, uterine/vaginal prolapse, rectocele, and anismus, with excellent concordance with clinical examination and POP-Q [24]. TPUS can also be used as a triage test in patients with symptoms of ODS, demonstrating high specificity and sensitivity for visualizing the extent of rectocele, uterine/vaginal prolapse, and anismus [25].

The reference point for assessing the position of pelvic organs is a line drawn horizontally along the inferior margin of the pubic bone. Normally positioned pelvic organs lie above or on this reference line during the Valsalva maneuver. The extent of descent of each organ can be measured; however, this is not essential, as the primary purpose of TPUS is to detect or exclude abnormalities. For example, pathological anorectal descent can be diagnosed sonographically when the ARJ is seen 10 mm below the symphysis pubis. Its paradoxical movement (upward instead of downward during the Valsalva maneuver) is pathognomonic for anismus. Table 4 illustrates common pelvic floor pathologies identified on ultrasound.

**Table 4:** Ultrasound features of pelvic floor pathologies

	Description	Illustration
Cystocele	It is identified on US as the bladder descent > 10 mm below the inferior margin of the pubic bone.	TPUS image: the white line represents the reference line drawn along the inferior margin of the pubic bone. The yellow line illustrates the descent of the bladder base below the pubic bone (extent of cystocele). B, bladder.
Assessment of normal female urethra	The urethra is divided into three anatomical segments: (1) Intramural (proximal) urethra, corresponding to the bladder neck region. (2) Mid-urethra, which contains the rhabdosphincter (RS) and represents the main continence mechanism. (3) Distal urethra, extending toward the external meatus.	EVUS midsagittal (**A**) and coronal images (**B**) illustrating the zonal anatomy of the female urethra. BN, bladder neck; RS, rhabdosphincter; SP, symphysis pubis.
Urethral hypermobility	Increased bladder neck mobility, measured as the difference in bladder neck position between rest and Valsalva (e.g., > 10–15 mm displacement).	TPUS image showing the bladder neck distance (blue line) at **A** at rest and **B** at Valsalva.
Mid-urethral sling assessment	The sling appears as a hyperechoic band located in the correct position at the level of the mid-urethra, with symmetrical arms.	**A** TPUS coronal view and **B** axial 3D TPUS illustrating the normal positioning of a TVT tape (arrows). **C** EVUS midsagittal view, the dotted line indicating the urethral lumen with the arrow pointing at the normal mid-urethral location of the tape. **D** EVUS in a different patient with failed surgery showing distal displacement of the tape (yellow arrow).
Uterine descent	Cervix at or below the inferior margin of the pubic bone.	TPUS showing the cervix (C) at the level of the inferior aspect of the pubic bone (SP). B, bladder.
Puborectalis muscle tears	Unilateral or bilateral detachment of the puborectalis muscle from the pubic bone or reduced muscle thickness. May be graded as partial or complete avulsion.	Axial images of the levator hiatus: **A** EVUS and **B** on 3D TPUS showing minor avulsion of the right puborectalis muscle (arrows).
Normal anal sphincter and anorectal status	Ultrasound using different probes can accurately assess the anatomy of the anal sphincter and lower rectum, which is essential for evaluating sphincter tears in patients with FI.	EVUS image acquired with the biplane probe facing posteriorly, illustrating a normal anal sphincter at rest.
Rectocele	Bulging of the anterior rectal wall with maintained continuity of the rectal wall within the prolapsing segment (> 15 mm).	TPUS showing the anterior rectocele extending beyond the expected contour of the anterior rectal wall during straining. AC, anal canal; B, bladder; PR, puborectalis; R, rectum; U, urethra.
Anorectal descent	Anorectal descent is identified when the ARA descends below the inferior margin of the pubic bone during straining.	TPUS showing the ARA below the pubic bone (horizontal reference line). The yellow line represents the degree of descent. B, bladder.
Rectal intussusception	Widening of the rectal lumen with inward folding (infolding) of the rectal wall into the lumen.	EVUS of the posterior compartment during Valsalva in a patient with enterocele (E) and rectal intussusception (IN). R, rectum.
Enterocele	Identified when small bowel loops herniate between the anorectum and vagina or (between rectum and bladder in post hysterectomy) during straining.	**A** TPUS during Valsalva showing bowel herniating between the rectum (R) and urethra (U). SP, symphysis pubis. **B** EVUS showing small bowel loops descending between the rectum and vaginal wall during Valsalva.

TPUS is also helpful in more clinically challenging cases, such as diagnosing enterocele or enterocele combined with rectocele. The presence of bowel loops between the anorectum and bladder is regarded as pathognomonic for enterocele. In combined cases, rectocele is clearly seen as bulging of the anterior rectal wall with continuity of the rectal wall throughout the prolapsing segment, whereas enterocele represents a prolapse from above, with the rectum and rectal wall visualized separately. Similarly, intrarectal intussusception may appear as luminal widening with enfolding of the rectal walls. When a definite diagnosis of cul-de-sac hernia or intussusception is limited by bowel gas artefacts, further imaging is recommended (MRD or FD if available).

Another role of TPUS is in patients with urinary incontinence (UI). Measurement of the bladder–symphysis distance (BSD), defined as the distance from the bladder neck to a line drawn horizontally along the inferior margin of the pubic bone, is a highly sensitive parameter for assessing the severity of UI. A difference of at least 10 mm in BSD between rest and Valsalva is considered the diagnostic threshold for urethral hypermobility.

TPUS can also be performed using a volumetric transducer (3D TPUS), which, in addition to 2D imaging, enables tomographic or multislice imaging in the axial plane. Compared with 2D TPUS, its main advantage is the ability to assess the entire puborectalis muscle and levator hiatus (LH), and to identify potential defects or detachment from the pubic rami, a known factor for recurrence of POP. Biometric indices of the LH, including area, length, and width, as well as puborectalis muscle thickness, can be measured with excellent interobserver reliability and high concordance with MRI [3, 26]. Patients with a larger hiatal area are more prone to developing POP and, if this is associated with levator ani muscle (LAM) avulsion, are at higher risk of surgical failure. When real-time acquisition of volumetric 3D ultrasound data is performed (4D TPUS), it allows the determination of the degree of LH distension during Valsalva and more confident visualization of LAM avulsion during muscle contraction. However, there is no superiority of 4D TPUS over 2D TPUS in the diagnosis of POP [27].

### Endovaginal US

EVUS is performed using a dedicated biplane mechanical transducer with 3D automatic acquisition to evaluate the pelvic floor according to a predetermined protocol in sagittal and axial sections [28]. EVUS of the anterior compartment enables detailed assessment of the urethra and its anatomy, periurethral/vaginal masses with the use of color Doppler, allowing for differentiation of soft-tissue masses and determining the vascularity of the urethra, which could be useful in cases of urinary incontinence.

Automatic 3D acquisition gives an overall view of the pelvic floor in the axial plane. 3D cube data can be reformatted in any desirable plane and angle, allowing for precise assessment of the LH and LAM with its subdivisions. 3D EVUS has shown strong agreement for LAM avulsion and LH measurements as compared to MRI and TPUS [29].

### Endoanal US

EAUS is considered the reference standard for anal sphincter evaluation in FI [4]. Three-dimensional acquisition assesses the anal canal from the upper aspect of the puborectalis muscle to the anal verge, in three levels: (1) proximal (deep): at the most cranial level of the puborectalis; (2) mid (superficial): IAS and EAS forming complete rings; (3) distal (subcutaneous): only EAS is visualized [3]. Normal EAS appears as a thick (approximately 10 mm) hyperechoic external ring, while IAS is a thinner internal hypoechoic ring. Any defects appear as a disruption of those rings, and their severity is measured, given the percentage of the thickness of the muscle bulk affected and its extent, using clockface notation [3]. EAUS and MRI can be used interchangeably for the detection of anal sphincter tears; however, when available, EAUS is the preferred modality for assessing sphincter integrity in FI (Supplementary Fig. S1), as it demonstrates superior sensitivity for detecting internal anal sphincter tears and sensitivity comparable to MRI for external anal sphincter injuries [30].

## Postoperative imaging of the pelvic floor

Management of PFD typically begins with conservative measures, including pelvic floor physiotherapy to strengthen pelvic floor muscles and biofeedback therapy to improve neuromuscular coordination. In patients where conservative treatment fails, or in cases when symptoms such as incontinence significantly impair quality of life, or in the presence of overt prolapse, surgical intervention may be indicated, depending on the affected pelvic compartment and the underlying pathology. For POP reconstructive procedures include sacrocolpopexy or sacrohysteropexy, sacrospinous fixation, and native tissue pelvic floor repair with or without mesh placement [31, 32]. Management of SUI may involve surgical options such as mid-urethral sling procedures, including the retropubic tension-free vaginal tape (TVT) and the transobturator tape (TOT), colposuspension, and autologous fascial slings. Periurethral bulking agents may also be used, particularly in selected patients with intrinsic sphincter deficiency or those unfit for more invasive surgery. However, the use of synthetic mid-urethral slings such as TVT and TOT has declined in recent years due to concerns regarding mesh-related complications. For FI, surgical management includes implantation of sacral nerve stimulation (SNS) while in patients with sphincter injury, anal sphincteroplasty is performed [33, 34]. In the posterior compartment, procedures such as rectopexy may be performed in patients with rectal prolapse, while stapled trans-anal rectal resection (STARR) can be performed in ODS patients with rectocele and/or rectal intussusception.

Awareness of these surgical techniques and implanted materials is essential for radiologists to accurately assess postoperative anatomy, recognize expected postoperative appearances, and detect complications. The choice of imaging modality in the postoperative setting depends on the clinical indication. CT is typically used in the early postoperative period to evaluate suspected hematoma, abscess, or bowel obstruction [35]. TPUS or EVUS are commonly used to assess mid-urethral sling position and related complications [31]. Static MRI provides excellent soft-tissue contrast and enables evaluation of mesh position, pelvic support structures, and postoperative complications. Thin-section T2-weighted imaging or 3D SPACE sequences can help delineate the location of implanted mesh or slings, while intravenous gadolinium with fat-suppressed T1-weighted sequences is valuable for detecting complications such as abscesses [32]. Dynamic MRD or FD can assess functional outcomes and identify recurrent prolapse, rectocele, enterocele, or rectal intussusception following surgery.

Normal postoperative appearances vary according to the surgical technique. Mid-urethral slings typically appear as a linear structure beneath the mid-urethra with a characteristic U-shaped configuration on ultrasound or MRI. Following sacrocolpopexy, the mesh may be identified as a thin linear structure extending from the vaginal vault to the sacral promontory and usually demonstrates low signal intensity on MRI (Supplementary Fig. S2). In patients with SNS implantation, the electrode is typically positioned within the S3 neural foramen, with the pulse generator located in the subcutaneous tissues of the gluteal region. Plain radiographs are useful for confirming appropriate electrode positioning (Supplementary Fig. S3).

### Summary statement

Establishing a radiological pelvic floor service requires more than just appropriate infrastructure and access to imaging modalities; it relies on effective communication with patients and close collaboration with referring clinicians. A high-quality service depends on a skilled multidisciplinary team, clear communication with patients, and sensitivity to the intimate nature of these examinations. Continuous audit, rigorous reporting, and active multidisciplinary collaboration are essential to maintain high standards and ensure imaging remains clinically relevant.

### Patient summary

Radiological investigations play a central role in the evaluation of PFD by enabling assessment of the underlying structural and functional abnormalities. Accurate diagnosis and individualized management often require integration of physiological tests, such as anorectal manometry and urodynamic studies, with multimodality imaging, including fluoroscopy, MRI, and ultrasound. Together, these techniques provide a comprehensive evaluation of pelvic floor musculature, supporting connective tissues, and pelvic organ anatomy and function.

## Supplementary information

ELECTRONIC SUPPLEMENTARY MATERIAL

## Abbreviations

ARA: Anorectal angle
ARJ: Anorectal junction
EAS: External anal sphincter
EAUS: Endoanal ultrasound
EVUS: Endovaginal ultrasound
FD: Fluoroscopic defecography
FI: Fecal incontinence
IAS: Internal anal sphincter
ODS: Obstructive defecation syndrome
PCL: Pubococcygeal line
PFD: Pelvic floor dysfunction
PFM: Pelvic floor muscles
POP: Pelvic organ prolapse
TPUS: Transperineal ultrasound
