# Supplementary material for: ESR Essentials: pelvic floor imaging—practice recommendations by the European Society of Urogenital Radiology
Source: Eur Radiol. 2026 Jul 1;36(11):8422–39. doi: 10.1007/s00330-026-12706-1 (PMC13661720; doi:10.1007/s00330-026-12706-1)
Supplement: Supplementary file 1 [file 330_2026_12706_MOESM1_ESM.pdf]

ESR Essentials: pelvic floor imaging-practice recommendations by the European Society of Urogenital Radiology

Electronic Supplementary Material

Table S1. Overview of contrast agents used for FD

**Organ**	**Type of contrast**	**Amount**	**How to administer?**	**When?**
Rectum	150 mL of Barium Sulphate mixed with 400 mL of water	120-240 mL (Until the patient experiences an urge to defecate)	Via rectal tube or catheter tip syringe	Immediately before the examination
Small bowel	Diluted Barium Sulphate (oral suspension)	180 mL of water added to one pot of E-Z-HD	Oral ingestion	1-2 hours before the examination
Vagina	Iodine-based contrast medium, with water-soluble gel and food thickening agent to form a wallpaper paste consistency	5-30 mL	Via catheter tip syringe	Immediately before the examination

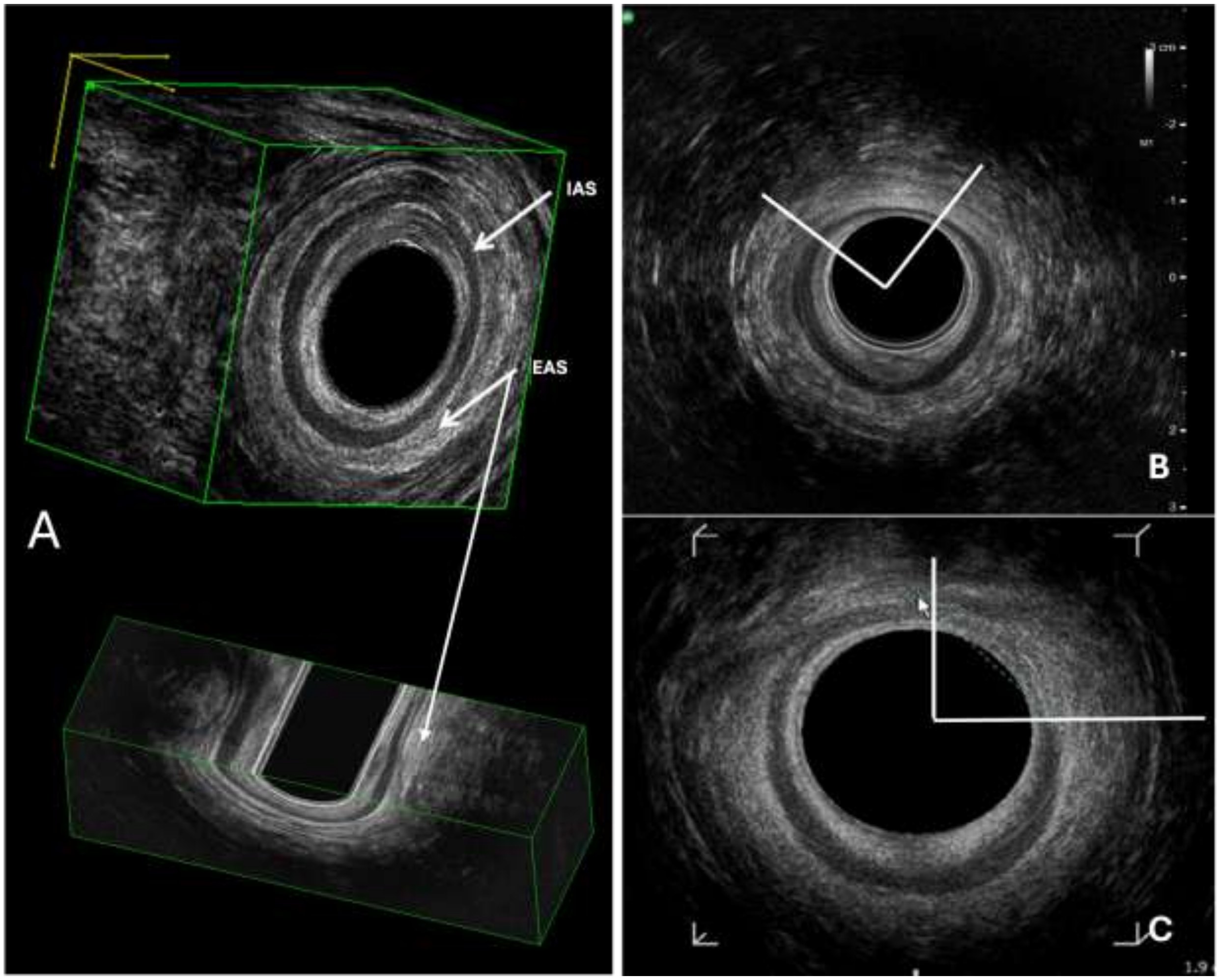

Fig. S1: EAUS in a female patient with grade 3 perineal tear presenting with faecal incontinence. **(A)** 3D cube acquisition of the anal canal in the axial plane (top) and coronal reformatting (bottom). Cube acquisition can be reformatted to different planes to assess sphincter integrity. **(B)** axial image illustrating the EAS injury with disruption of the outer hyperechoic ring anteriorly from 10 to 1 o'clock position. **(C)** axial image showing IAS injury with discontinuity of the inner hypoechoic layer at the left anterolateral quadrant (12 to 3 o'clock position).

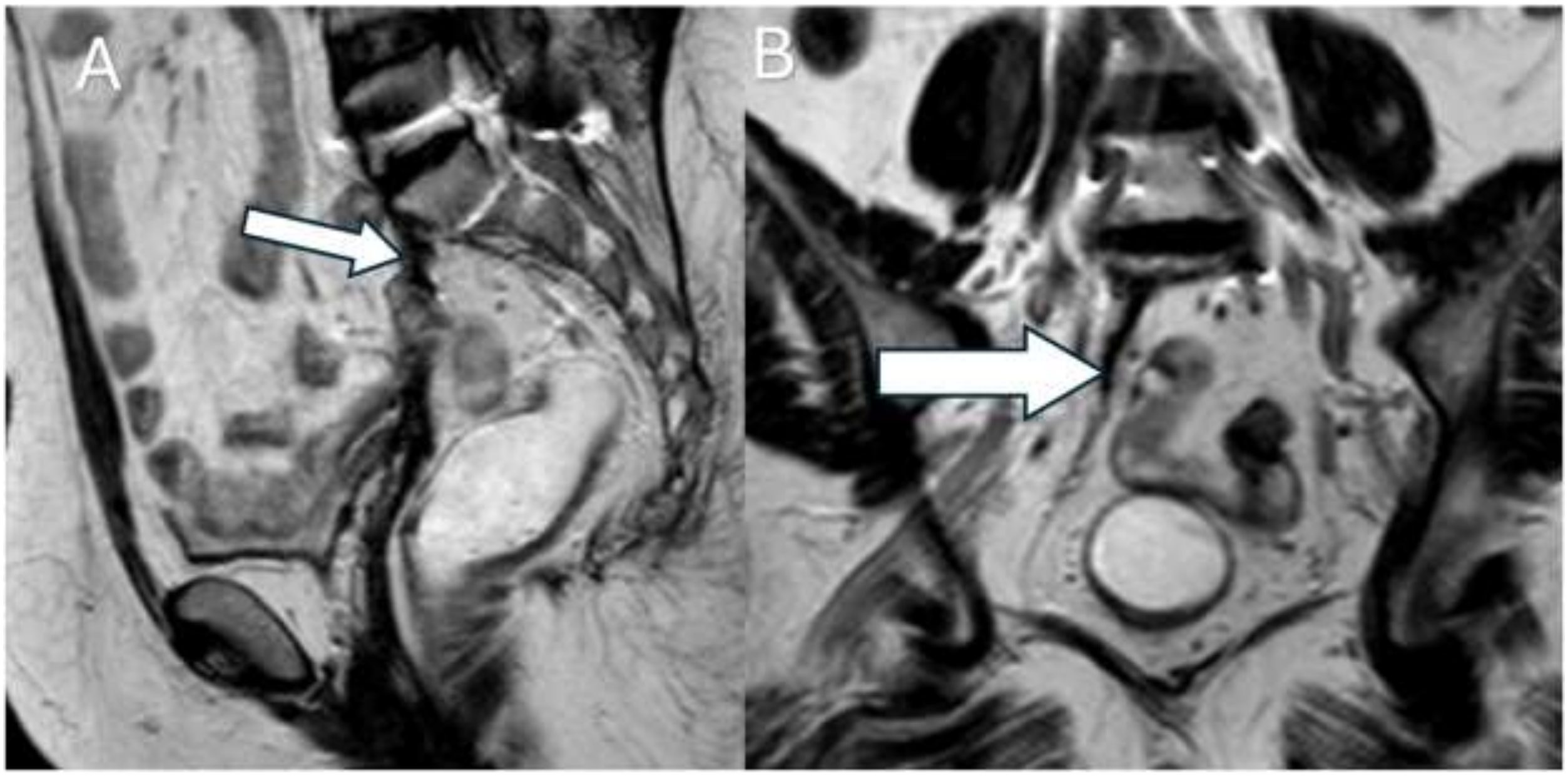

Fig. S2: **(A)** Sagittal T2 and (**B)** coronal T2 static MRI images showing the appearance of mesh sacrocolpopexy in a 65-year-old for treatment of vaginal prolapse post hysterectomy.

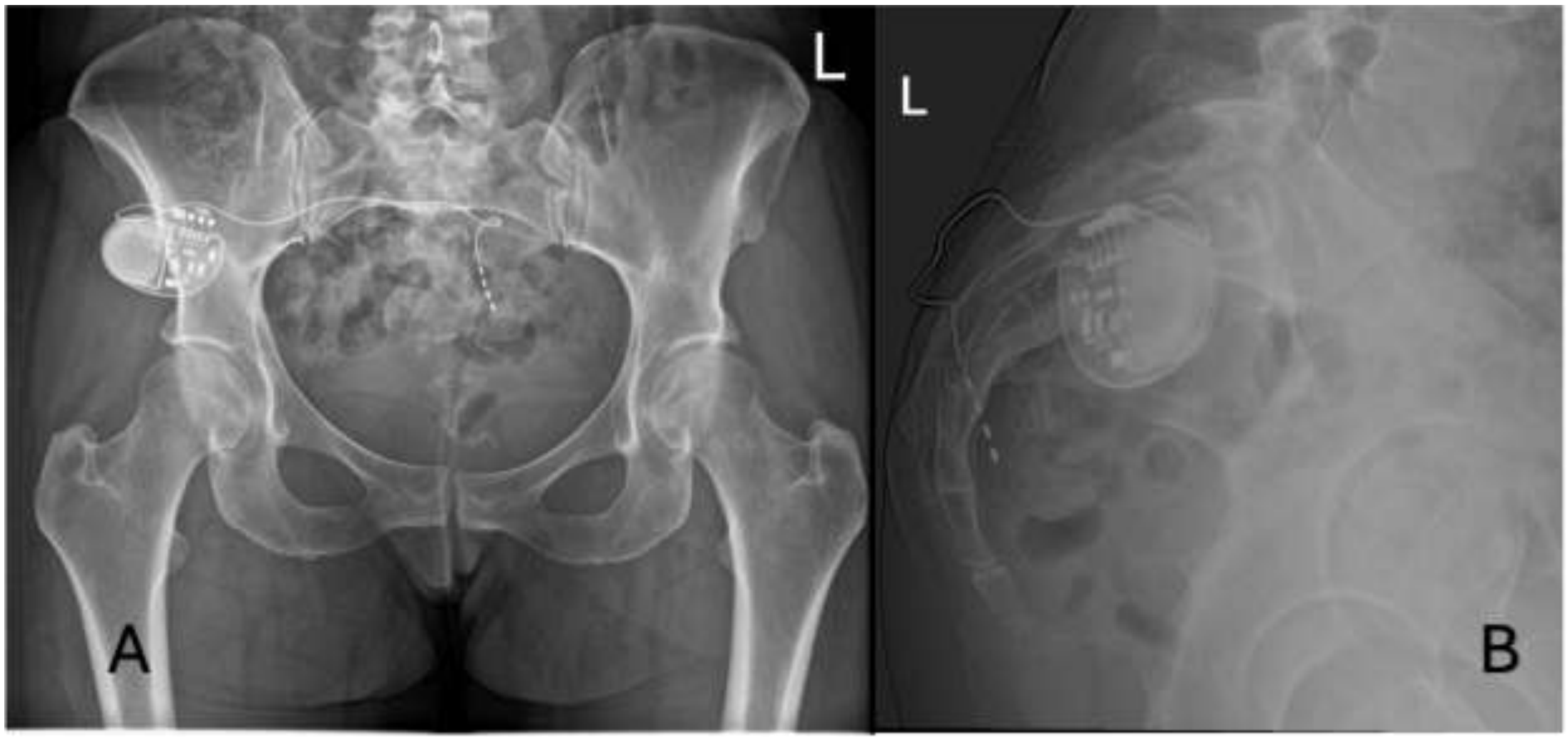

Fig. S3: (A) AP and **(B)** lateral plain radiographs of the sacrum illustrating the normal positioning of an implanted sacral nerve stimulator (SNS) in a 47-year-old female patient complaining of faecal incontinence. The lead appears in situ with no evidence of disconnection, fragmentation or complications.
